# Biodegradable polyester-based hyperbranched nanocarrier-modified with N-acetyl glucosamine for efficient drug delivery to cancer cells through GLUTs

**DOI:** 10.3389/fbioe.2025.1491206

**Published:** 2025-02-28

**Authors:** Aazam Shaikh, Rajesh Salve, Devyani Sengar, Virendra Gajbhiye

**Affiliations:** ^1^ Nanobioscience Group, Agharkar Research Institute, Pune, India; ^2^ Savitribai Phule Pune University, Pune, India

**Keywords:** glucose transporters, doxorubicin, breast cancer, Dendritic nanocarrier, H40 Boltorn, drug delivery

## Abstract

Cancer, ranking just below cardiovascular diseases, is a leading cause of mortality worldwide. The key to enhancing survival rates among cancer patients lies in the early detection, removal, and treatment of tumors. However, the broad-spectrum nature of current treatments, including chemotherapy and radiation therapy, results in significant collateral damage to healthy cells and tissues. In this context, hyperbranched polymers present a promising avenue for more targeted therapy. These polymers can be loaded with chemotherapeutic drugs and modified with specific ligands to selectively target cancer cells via glucose transporters, which are overexpressed in many cancer types. To enhance the delivery of drugs to cancer cells, we have engineered an N-acetyl glucosamine conjugated version of this polymer. The characterization of these nanocarriers was evaluated using various techniques, including ^1^H NMR, dynamic light scattering, and FTIR spectroscopy. Additionally, confocal microscopy was utilized to compare the accumulation of doxorubicin in cancer cells using both the N-acetyl glucosamine-conjugated and unmodified versions of H40 Boltorn™. Our observations indicated a superior accumulation of doxorubicin in cells treated with the modified H40 polymer. Further evaluation of the drug-loaded nanocarriers was conducted on MDA-MB-231 and 4T1 breast cancer cell lines, focusing on their cytotoxic effects. This suggests that the targeted delivery of anticancer drugs using the modified H40 Boltorn™ nanocarriers significantly enhances the ability to kill breast cancer cells, offering a more efficient and selective approach to chemotherapy that minimizes impact on healthy tissues and cells.

## 1 Introduction

As we know it today, chemotherapy was first coined by Paul Ehrlich in the early 20th century but came into practice after the Second World War. During the war, it was observed that soldiers exposed to mustard gas had decreased leukocyte cell counts, suggesting the use of alkylating agents for the treatment of lymphomas. The late 1950s saw the advent of combination chemotherapy by Emil Frei III for the treatment of acute lymphoblastic leukemia, resulting in lasting remissions and, in some cases, curing the cancer (Rosenthal, 2013). Combination chemotherapy is now a standard therapeutic approach in treating many adult and pediatric cancers (Arruebo et al., 2011; Sodergren et al., 2020; Tiwari et al., 2022). Since then, numerous compounds have been chemically synthesized or discovered for cancer treatment. However, all these compounds work on the principle of indiscriminately killing cells, which damages circulating immune cells and other healthy cells and tissues. The clearance of these compounds from circulation may cause accumulation in other susceptible and vital organs such as the liver, kidney, spleen, and heart. Moreover, higher dosages of these compounds are administered to reach toxic concentrations in the tumor environment, thus increasing therapy costs. Chemotherapy is one of the most fundamental therapies, recommended along with radiation therapy, after the surgical removal of the tumor. Approaches such as immunotherapy use antibodies to target cancer cells, and they are much more efficient than using only cytotoxic drugs. However, immunotherapy is unaffordable to most people. Another approach to target cancer cells is utilizing specific receptors and transporters overexpressed on these cells (Byrne et al., 2008).

The use of nanoparticles (NPs) to deliver chemotherapeutic drugs has been ever-evolving for the past two decades (Kaushik et al., 2022). NPs can be used as drug carriers, modified with ligands crucial in targeting cancer cells (Yan et al., 2024). Xu et al. have reported the synthesis of aptamer-conjugated polyester dendrimer conjugates for the targeted delivery of doxorubicin (DOX), specifically in prostate cancer cells (Xu et al., 2013b). Aptamers bind to cell receptors to facilitate cellular entry. There should be a high number of receptors on the cell for high cellular uptake of drugs through this method, or rapid receptor recycling is required for drug accumulation in the cell. In contrast, uptake through transporters is constant and does not require recycling.

Glucose Transporters (GLUTs) play a critical role in cell survival as they are responsible for absorbing glucose into the cell from the bloodstream (Rivlin and Navon, 2016). Furthermore, GLUT overexpression increases malignancy and the ability of cancer cells to metastasize and causes high mortality, reducing survival rates (Pliszka and Szablewski, 2021). Similarly, other studies have shown that NAG conjugation results in higher cellular uptake of nanoparticles (Kumar et al., 2017a; Kumar et al., 2018a; Pooja et al., 2020). Nanoparticles can be engineered to be stimuli-responsive and have a controlled release, increasing the effectiveness of the treatment (Salve et al., 2021). In various studies, NPs can remediate multi-drug resistance and reduce off-target effects. In pursuit of designing completely biodegradable and pH-responsive NPs that can deliver the drug effectively to cancer tissues, we chose polyester-based H40 Boltorn hyperbranched NPs (Carlmark et al., 2013; Zhang et al., 2014). H40 Boltorn is a commercially available hyperbranched polymer that has been widely used for anticancer theranostic studies (Korake et al., 2021). Moreover, H40-Boltorn is a polyester-based nanocarrier that is highly sensitive to acidic pH conditions, which causes it to degrade. This characteristic allows H40-Boltorn to release its cargo in acidic environments. The relationship between cancer and glucose consumption, known as the Warburg effect, has been extensively studied. Research indicates that the cancer microenvironment typically has a slightly more acidic pH compared to normal tissues. In this study, hyperbranched H40 Boltorn was modified with N-acetyl glucosamine (NAG) and loaded with anticancer drug doxorubicin to enhance its transport into the cancer cells through glucose transporters (GLUTs).

## 2 Methods

### 2.1 Synthesis and characterization

**FIGURE 1:**
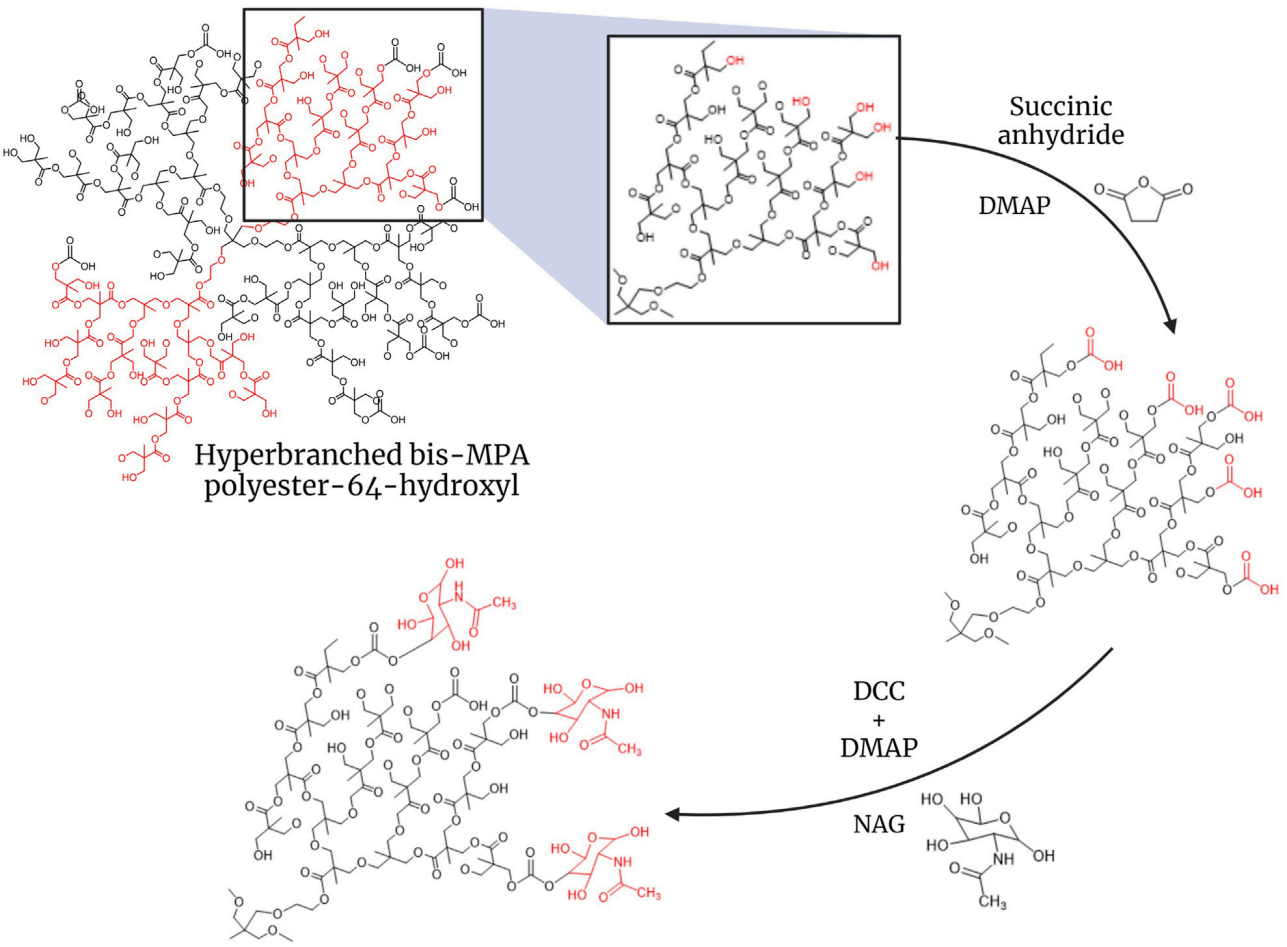
Synthesis of H40-NAG hyperbranched polymer for targeting GLUTs.

A quantity of H40 equivalent to 10 µmol was dissolved in DMSO. H40 Boltorn has terminal hydroxy groups, which were carboxylated using succinic anhydride in the presence of DMAP (Figure 1) (Gajbhiye et al., 2014). First, succinic anhydride was dissolved in a small amount of DMSO. Once fully dissolved, DMAP was added. The H40 solution was added, and the mixture was stirred on a magnetic stirrer for 30 min before adding 1 mL of tetrahydrofuran (THF). It was left overnight under inert conditions at room temperature. The reaction mixture was then added to chilled diethyl ether dropwise to precipitate it and was incubated at −20°C overnight. The precipitate was recovered via centrifugation (at 12,000 rpm, for 30 min at room temperature). The obtained pellet was dried by lyophilization.

To conjugate NAG, H40-COOH was first dissolved in DMSO. The solution was then subjected to N_2_ gas bubbling to create an inert atmosphere. DCC and DMAP were added to H40-COOH, and the reaction was carried out on ice (Kumar et al., 2017b). The mixture was then incubated on a magnetic stirrer for 2 h at room temperature. NAG was dissolved in DMSO and added to the reaction mixture. The entire mixture was incubated for 48 h at room temperature on a magnetic stirrer. The conjugated NPs were then dialyzed against water with regular water changes, using a 3.5 kDa cut-off membrane for 24 h. After dialysis, the NPs obtained were lyophilized. The lyophilized NPs were then analyzed using Dynamic Light Scattering (DLS) in MilliQ water with (replication number, n = 3) to determine changes in the size and charge of the nanocarrier. FTIR spectroscopy and ^1^H NMR were done to assess the conjugation of NAG on the nanocarrier.

### 2.2 Drug loading

To load DOX, 40 mg of the drug was weighed and dissolved in 400 µL of DMSO. Separately, the nanocarrier (20 mg) was weighed and dissolved in 500 µL of DMSO. DOX was then mixed dropwise with the nanocarrier, followed by adding 100 µL of DMSO to the tube to recover the remaining DOX and make the final volume 1 mL. The solution was stirred on a magnetic stirrer for 48 h, followed by dialysis for 2 h using a 3.5 kDa membrane. UV–vis spectroscopy was used to assess the quantity of drug in the supernatant (Xu et al., 2013a; Kumar et al., 2018b). The amount of drug loaded in the nanocarrier was calculated by establishing the difference between the DOX used first and the DOX in the supernatant. The loaded drug has been reported as weight % loading per mg of nanocarrier. The membrane content was vacuum-dried after dialysis to obtain DOX-loaded H40 nanocarrier for further studies.

### 2.3 Cellular uptake

MCF7 and 4T1 breast cancer cells were plated as 1 × 10^5^ cells per well in a six-well plate. Cellular uptake was also carried out on NIH-3T3 non-cancerous cells in similar numbers separately to evaluate the specificity of drug-loaded nanocarriers. The cells were then incubated at 37°C with 5% CO_2_ for 24 h. Following the incubation, the cells were exposed to free DOX, DOX-loaded H40-OH, and DOX-loaded H40-NAG to examine their cellular uptake. After 2 h, the media containing the drug and nanocarriers was removed, and the cells were washed with PBS. The cells were then fixed using 3.75% PFA for 10 min and washed with PBS thrice. Then, the cells were stained with phalloidin-Alexa488 for 30 min and nuclear stain DAPI for 10 min. After staining, the cells were washed and mounted on slides using glycerol as the mounting medium (Salve et al., 2024).

### 2.4 Cytotoxicity

MCF7 and 4T1 cells were seeded in 96-well plate at a concentration of 1 × 10^4^ cells per well. The MCF7 and 4T1 cells were treated at 0.05, 0.5, 5, and 50 µM concentrations of DOX in the treatment groups of free-DOX, H40-OH + DOX, and H40-NAG + DOX in serum-free medium. The cells were then incubated for a period of 24 h and 48 h. After the incubation, MTT end point assay was performed to determine cell viability.

### 2.5 Cell death and apoptosis

A 24-well plate was seeded with 1 × 10^5^ 4T1 cells per well to estimate the cell death and apoptosis population upon treatment with DOX, H40+DOX, and H40-NAG + DOX. The cells were treated after 24 h of seeding and incubated for further 12 h. The cells were then trypsinized and centrifuged. The cells were stained as per the manufacturers’ instruction using AnnexinV/PI.

### 2.6 Statistical analysis

Cytotoxicity data was analyzed by two-way analysis of variance (ANOVA), and the data was compared using Dunnett’s multiple comparison-test in GraphPad Prism statistical software (GraphPad, version 9.5.0).

## 3 Results

### 3.1 Characterization of the nanocarriers

**FIGURE 2:**
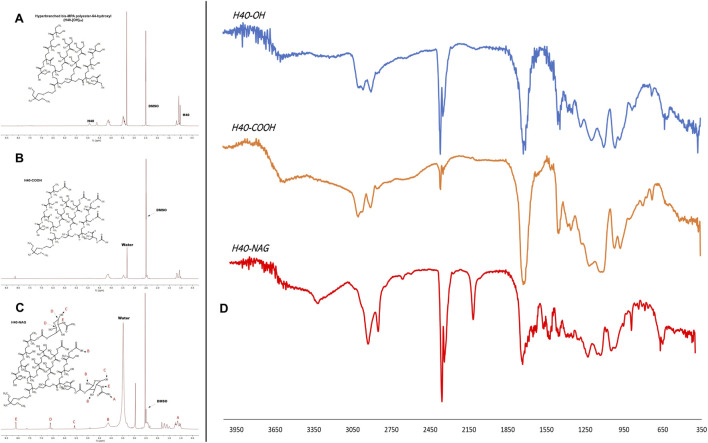
NMR spectrum of **(A)** Unmodified H40, **(B)** H40-COOH, **(C)** NAG-modified H40 nanocarrier, and **(D)** FTIR spectrum of H40-OH, H40-COOH, H40-NAG.

The size of the unmodified generation 4 (G4) H40-OH hyperbranched polymer was found to be around 43.82 ± 11.30 d nm. (Supplementary Figure 1A). After modification with succinic anhydride and functionalization of the carboxyl group, the size of the nanocarrier increased to 58.77 ± 7.04 d nm (Supplementary Figure 1C). The conjugation of N-acetyl glucosamine (NAG), depicted an increase in size to 96.08 ± 6.79 d nm (Supplementary Figure 1E). The zeta potential of unmodified H40-OH was −13.06 ± 3.24 mV, which increased to −28.3 ± 8.03 mV, whereas that of H40-NAG was 5.21 ± 1.70 mV (Supplementary Figures 1B–F respectively). NMR of H40, H40-COOH, and H40-NAG were recorded in DMSO on Bruker Advance III HD NMR 500 MHz spectrometer (Istratov et al., 2021). Characteristic peaks of N-acetyl glucosamine can be observed at 8.11, 6.63, 5.58, and 4.12 ppm (Figures 2A–C). The conjugation of H40-COOH with N-acetyl glucosamine was also verified using Fourier transform infrared (FTIR) spectroscopy. As shown in Figure 2D, the spectrum is of H40-OH (top), H40-COOH (middle) and the lower spectrum is of H40-NAG. The region highlighted around 800 cm^−1^ depicts the -NH wag or presence of the amine group of NAG and is absent in the upper spectra. Similarly, the region around 1700 cm^−1^ depicts C=O stretching. Lastly, the region highlighted around 3000 cm^−1^ demonstrates O-H stretching in carboxylic acids, having a variable and broad nature.

Drug loading in a nanocarrier is achieved by creating a high concentration gradient and a low concentration. The drug loaded in the nanocarrier showed 16.33 ± 7.42 wt% loading in the ratio of 1:2 in unmodified H40. On the other hand, H40-NAG showed 32.11 ± 0.53 wt% loading in the ratio of 1:2. This difference is due to H40-NAG having higher drug loading than H40-OH because the terminal ends of the hyperbranched polymer modified with the sugar moiety (NAG) act as terminal barriers and restrict the diffusion of the drug.

### 3.2 Cellular uptake

**FIGURE 3:**
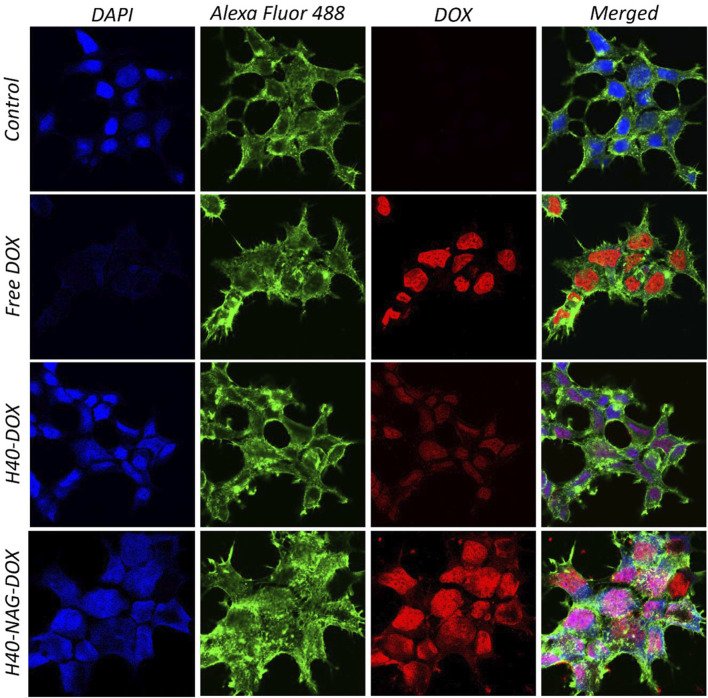
Confocal images showing cellular uptake of DOX loaded -H40 and -H40-NAG in MCF7 cells.

MCF7 cells displayed significant binding of free DOX (Figure 3), specifically in the nuclear region, while H40-OH and H40-NAG showed distribution throughout the entire cell. Moreover, the fluorescence intensity of DOX was notably higher in H40-NAG than in H40-OH. The higher fluorescence indicates increased accumulation or cellular uptake of H40-NAG-loaded DOX. Free DOX exhibited the highest mobility and accumulation in the nucleus due to its lower molecular weight and size compared to H40-OH + DOX and H40-NAG + DOX.

In the Free DOX panel, DOX is distinctly localized to the nucleus due to its nature to bind with double-stranded DNA. The distinction in the quantity of DOX delivered by unmodified H40 and H40 modified with NAG can be observed by comparing the fluorescent intensity of DOX in the respective groups. The H40-NAG + DOX exhibits higher cellular uptake and fluorescent intensity of DOX, as a higher number of modified nanocarriers are taken up by the GLUTs present in the cancer cells for glucose uptake.

Similarly, the uptake of drug-loaded nanocarriers was studied in NIH-3T3 and 4T1 cells (Supplementary Figure 4, 5, respectively). In the healthy NIH-3T3 cells, free-DOX significantly shows high accumulation in the nucleus in comparison with H40-OH and H40-NAG nanocarriers. In contrast, the cancerous 4T1 cells show high accumulation of free-DOX as well as H40-NAG nanocarriers, demonstrating that H40-NAG nanocarriers have higher uptake by cancer cells. This aligns with the previous cellular uptake experiment in MCF7 and cell death assessed using flow cytometry.

### 3.3 Cytotoxicity

Following evaluation of the cellular uptake, the nanocarriers’ ability to induce cell death through drug delivery was confirmed using the MTT assay. In the human breast cancer cell line MCF7, unmodified H40 exhibited the lowest activity, while Free DOX and H40-NAG + DOX demonstrated significantly higher cell death at 24 and 48 h (Supplementary Figure 6). Similarly, the 4T1 cell line also displayed significantly higher cell death at 48 h compared to 24 h, at all concentrations. It was demonstrated that the nanocarriers effectively delivered DOX, as H40-NAG + DOX resulted in 26.98% ± 2.01% cancer cell viability, compared to 34.52% ± 2.81% for DOX alone, and 49.04% ± 1.62% for H40-OH + DOX in the MCF7 cell line after 48 h of treatment with 50 µM DOX. Similarly, in 4T1 cells, the viability was 17.56% ± 5.17%, 30.30% ± 2.62%, and 48.24% ± 2.23% for H40-NAG + DOX, free-DOX, and H40-OH + DOX, respectively. Thus, this affirms the ability of H40-NAG to efficiently deliver DOX, resulting in cell death.

### 3.4 Cell death and apoptosis

**FIGURE 4:**
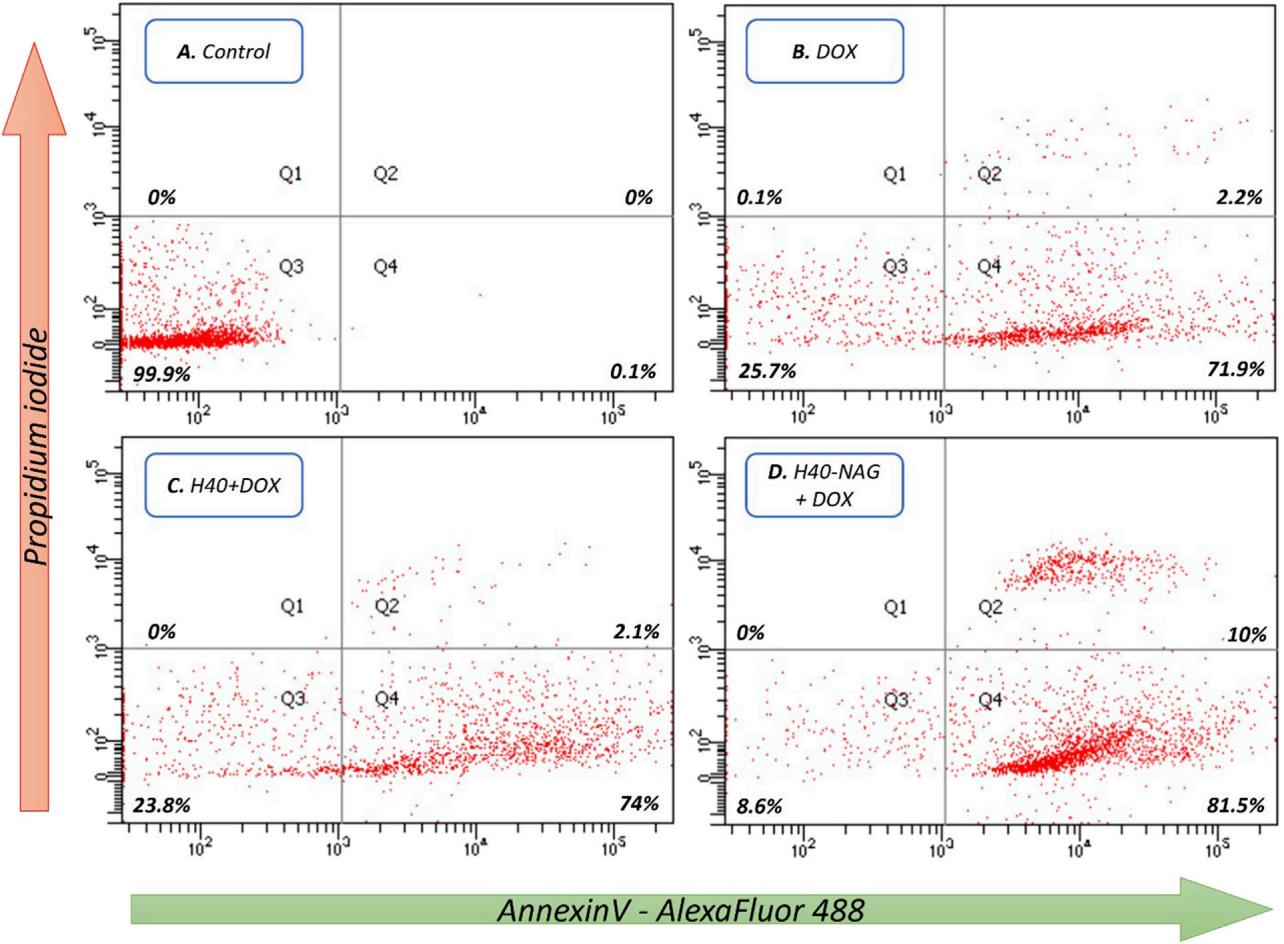
Flow cytometric analysis of 4T1 cells as follows **(A)** Control, **(B)** DOX, **(C)** H40 + DOX, and **(D)** H40-NAG + DOX.

The Annexin V/PI dual staining approach has been leveraged to quantitatively measure apoptosis. A hallmark of apoptosis is the increased availability of phosphatidylserine (PS) on the cell’s outer membrane, which becomes detectable through fluorescence when Annexin V molecules, tagged with fluorescent labels, are applied. Concurrent staining with propidium iodide (PI) highlights dead cells. This dual staining technique effectively differentiates between viable, early apoptotic, late apoptotic, and necrotic cells. In studies involving 4T1 cells, it was observed that hyperbranched polymers modified with NAG induced greater rates of cell death compared to their non-targeted counterparts. After 6 h at a concentration of 7.5 μg/mL, the formulations H40-NAG + DOX, H40-OH + DOX, and Free DOX demonstrated late apoptosis rates of 10%, 2.1%, and 2.2%, respectively (Figure 4). The overall population of cells undergoing apoptosis was found to be 91.5%, 76.1%, and 74.1% in H40-NAG + DOX, H40-OH + DOX, and free DOX-treated cells. Flow cytometry further verified the apoptosis-driven cell death in 4T1 cells due to DOX exposure.

## 4 Discussion

H40 Boltorn is a polyester-based hyperbranched nanocarrier that is entirely biodegradable at the lysosomal pH. The chemical modification of the H40 nanocarrier was studied at every step using FTIR, NMR, and DLS. H40-NAG conjugated nanocarriers demonstrated efficient drug loading in comparison with H40 nanocarriers. The drug release study (Supplementary Figure 3) showed higher drug release at acidic pH 5.5 compared to a physiological pH 7.4. Further analysis of drug release kinetics was done using DDSolver (Zhang et al., 2010). The release kinetics were observed as per the Korsmeyer-Peppas mathematical model, which describes drug release from polymeric systems. H40-NAG also showed significantly higher cellular uptake than H40-OH. Cancer cells have more GLUT transporters than normal cells, thus NAG modification allows for greater absorption by cancer cells. This was verified in the normal fibroblast cell line NIH-3T3, where free-DOX exhibited higher accumulation (Supplementary Figure 4), while in the cancerous cell line 4T1, both Free-DOX and H40-NAG showed significant accumulation (Supplementary Figure 5). Thus, the nanocarriers show higher uptake in cancerous cells than normal cells. Furthermore, the cytotoxicity of H40-NAG was also significantly higher when tested on 4T1 and MCF7 cells over 24h and 48 h. Cells undergoing cell death through apoptosis was confirmed by flow cytometry by dual staining of AnnexinV/PI. The dual staining confirmed apoptosis and showed cells undergoing late and early apoptosis. Significantly higher cells were undergoing late apoptosis in the H40-NAG nanocarrier-treated group, affirming their quicker and higher uptake. Thus, utilizing degradable nanocarriers like H40 Boltorn, laden with drugs, and cellular uptake enhancers like NAG could be highly successful as anticancer therapeutics.

## Data Availability

The original contributions presented in the study are included in the article/Supplementary Material, further inquiries can be directed to the corresponding author.
