# Supplementary material for: Biodegradable polyester-based hyperbranched nanocarrier-modified with N-acetyl glucosamine for efficient drug delivery to cancer cells through GLUTs
Source: Front Bioeng Biotechnol. 2025 Feb 28;13:1491206. doi: 10.3389/fbioe.2025.1491206 (PMC11906665; doi:10.3389/fbioe.2025.1491206)
Supplement: Supplementary file 1 [file DataSheet1.docx]

**Biodegradable polyester based hyperbranched nanocarrier-modified with N-acetyl glucosamine for efficient drug delivery to cancer cells through GLUTs**

**Aazam Shaikh^1,2^, Rajesh Salve^1,2^, Devyani Sengar^1,2^, Virendra Gajbhiye^1,2*^**

^1^Nanobioscience Group, Agharkar Research Institute, Pune – 411004, India

^2^Savitribai Phule Pune University, Pune – 411007, India

*** Correspondence:**Virendra Gajbhiye
(virendragajbhiye@aripune.org; cme_virendra@yahoo.co.in)

Supplementary Material

# Materials and Methods

## Materials

Hyperbranched bis-MPA polyester dendrimer (H40-OH, generation 4), Succinic anhydride, 4-N, N-dimethylaminopyridine (DMAP), and Doxorubicin were purchased from Sigma-Aldrich. The cell lines used in this study were obtained from the National Center for Cell Sciences (NCCS), Pune, India.

## Synthesis and Characterization

A quantity of H40 equivalent to 10 µmol was dissolved in DMSO. H40 Boltorn has terminal hydroxy groups, which were carboxylated using succinic anhydride in the presence of DMAP (Figure 1) (Gajbhiye et al., 2014). First, succinic anhydride equivalent to 1 mmol was dissolved in a small amount of DMSO. Once fully dissolved, DMAP equivalent to 10% weight of succinic anhydride was added. The H40 solution was added, and the mixture was stirred on a magnetic stirrer for 30 min before adding 1 mL of tetrahydrofuran (THF). It was left overnight under inert conditions at room temperature. The reaction mixture was then added to chilled diethyl ether dropwise to precipitate it and was incubated at -20°C overnight. The precipitate was recovered via centrifugation (at 12000 rpm, for 30 min at room temperature). The obtained pellet of H40-COOH was dried by lyophilization.

To conjugate NAG, H40-COOH was first dissolved in DMSO. The solution was then subjected to N_2_ gas bubbling to create an inert atmosphere. DCC and DMAP were added to H40-COOH, and the reaction was carried out on ice (Kumar et al., 2017). The mixture was then incubated on a magnetic stirrer for 2 h at room temperature. NAG was dissolved in DMSO and added to the reaction mixture. The entire mixture was incubated for 48 hours at room temperature on a magnetic stirrer. The conjugated NPs were then dialyzed against water with regular water changes, using a 3.5 kDa cut-off polyamide dialysis membrane for 24 h. After dialysis, the NPs obtained were lyophilized. The lyophilized NPs were then analyzed using dynamic light scattering (DLS) (Malvern Zetasizer ZSP, Malvern, Germany) to determine changes in the size and charge of the nanocarrier. FTIR spectroscopy using Shimadzu IR Affinity-1 Fourier Transform Infrared spectrophotometer, and ^1^H NMR using Bruker Avance III HD NMR 500 MHz spectrometer were done to assess the conjugation of NAG on the nanocarrier.

## Drug loading

To load DOX, 40 mg of the drug was weighed and dissolved in 400 µl of DMSO. Separately, the nanocarrier (20 mg) was weighed and dissolved in 500 µl of DMSO. DOX was then mixed dropwise with the nanocarrier, followed by adding 100µl of DMSO to the tube to recover the remaining DOX and make the final volume 1 mL. The solution was stirred on a magnetic stirrer for 48 h, followed by dialysis for 2 h using a 3.5 kDa cut-off polyamide dialysis membrane. UV–vis spectroscopy was used to assess the quantity of drug in the supernatant (Kumar et al., 2018). The amount of drug loaded in the nanocarrier was calculated by establishing the difference between the DOX used first and the DOX in the supernatant. The loaded drug has been reported as weight % loading per mg of nanocarrier. The membrane content was vacuum-dried after dialysis to obtain DOX-loaded H40 nanocarrier for further studies.

## Drug release and kinetics

Drug release was performed in a total volume of 10 ml in a tube incubated at 37℃. For assessing release in physiological and acidic conditions, the pH of medium used was 7.4 and 5.5, respectively. For drug release analysis, equivalent weight 125 µg DOX loaded nanocarriers H40-OH and H40-NAG were diluted in 200µl medium and sealed in a dialysis bag with membrane cut-off 3kDa. The dialysis bag was then put in the tube containing 9.8 ml medium of respective pH. At fixed time points, 600 µl samples were taken for spectrophotometric measurement and replaced with fresh medium. The spectrometry was performed immediately at 480nm after each time point.

## Cellular uptake

MCF7 and 4T1 breast cancer cells were plated as 1 × 10^5^ cells per well in a six-well plate. NIH-3T3 non-cancerous cells were taken in the similar numbers seperately. The cells were then incubated at 37°C with 5% CO_2_ for 24 h. Following the incubation of 24 h, the medium was removed, and the cells were exposed to free DOX, DOX-loaded H40-OH, and DOX-loaded H40-NAG to examine their cellular uptake. After 2 h, the media containing the drug and nanocarriers was removed, and the cells were washed with PBS. The cells were then fixed using 3.75% PFA for 10 minutes and subsequently washed with PBS three times. Then, the cells were stained with phalloidin-Alexa488 for 30 min and nuclear stain DAPI for 10 min. After staining, the cells were washed, mounted on slides using glycerol as the mounting medium and imaged using a confocal microscope (Leica Microsystems, Model TCS-SP8, Germany) (Salve et al., 2024).

## Cytotoxicity

MCF7 and 4T1 cells were seeded in 96-well plate at a concentration of 1 × 10^4^ cells per well. The MCF7 and 4T1 cells were treated at 0.05, 0.5, 5, and 50 µM concentrations of DOX in the treatment groups of free-DOX, H40-OH+DOX, and H40-NAG+DOX in serum-free medium. The cells were then incubated for a period of 24 h and 48 h. Then, 10 μL of MTT reagent (5 mg/mL) was added to each well and incubated for 4 hrs in the CO_2_ incubator. Formed MTT formazan crystals in the wells were dissolved in 200 μL of DMSO. The absorbance was measured at 570 nm using the BioTek Synergy plate reader (Yu et al., 2014).

## Cell death and apoptosis

A 24-well plate was seeded with 1 × 10^5^ 4T1 cells per well to estimate the cell death at 37°C in CO_2_ incubator. The cells were then treated with DOX, H40+DOX, and H40-NAG+DOX after 24 hours of seeding and incubated for further 12 hours. The cells were then trypsinized and centrifuged (1000 RPM, 10 mins) to remove media residues. Cells were resuspended in 100 μL Annexin binding buffer. Thereafter, cells were stained with Alexa Fluor 488 Annexin V and propidium iodide (FITC annexin V/dead cell apoptosis kit, molecular probe) for 15 min at room temperature. The volume of the suspension was made up to 500 μL with annexin binding buffer. Samples were analyzed by flow cytometer (BD FACS Calibur, BD Biosciences, USA) (Crowley et al., 2016).

## Statistical analysis

Cytotoxicity data was analyzed by two-way analysis of variance (ANOVA), and the data was compared using Dunnett’s multiple comparison-test in GraphPad Prism statistical software (GraphPad, version 9.5.0).

# Supplementary Figures and Tables

## Supplementary Figures

### Size and zeta

**
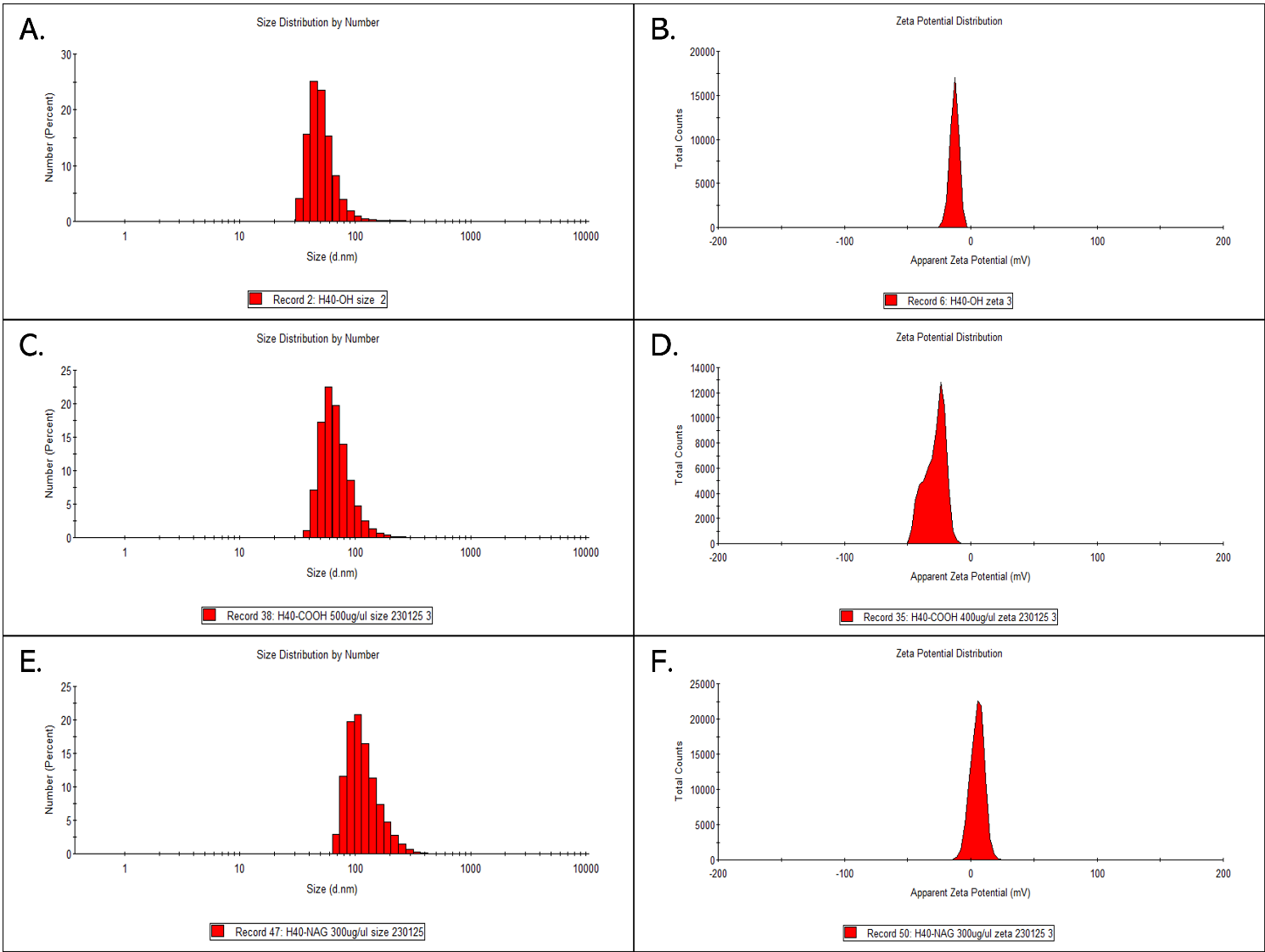
**

**Supplementary Figure 1.** Hydrodynamic radius and size of unmodified H40 Boltorn and N-acetyl glucosamine modified H40 (H40-NAG). (A) Average size of H40-OH, (B) Average zeta of H40-OH, (C) Average size of H40-COOH, (D) Average zeta of H40-COOH, (E) Average size of H40-NAG, (F) Average zeta of H40-NAG.

### Size of drug-loaded nanocarrier


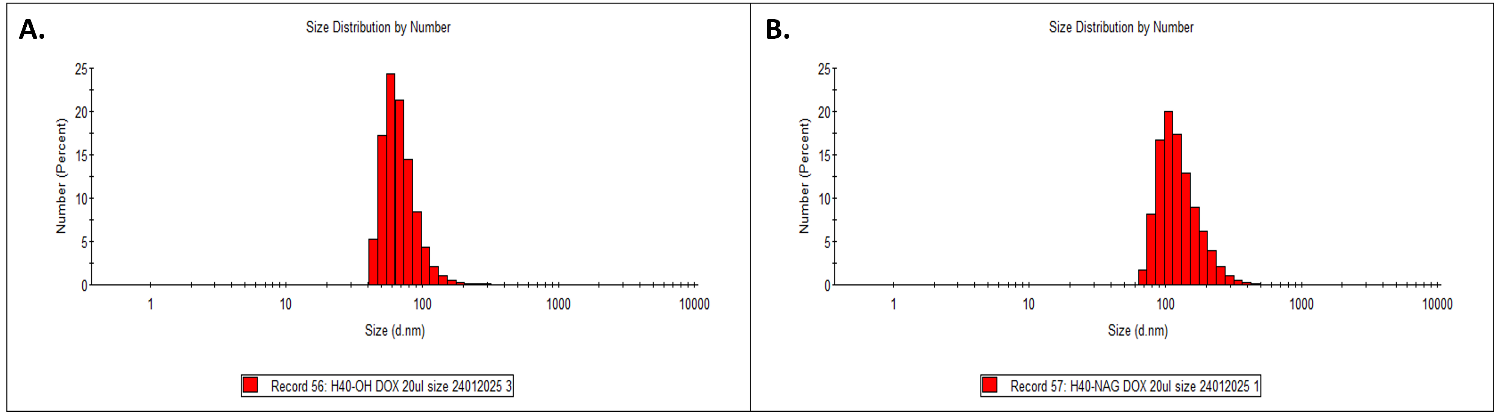


Supplementary Figure 2. DLS results showing size of (A) H40-OH+DOX and (B) H40-NAG+DOX.

The size of H40-OH+DOX and H40-NAG+DOX was measured using DLS, and the average size was found to be 58.77±10.47 d.nm and 105.7±18.85 d.nm. The size of the drug-loaded H40-NAG does not increase significantly in comparison with the non-loaded H40-NAG nanocarrier.

### Drug release study

The drug release data was analyzed using DDSolver and release kinetics models (Zero order, First order, Hixson-Crowell, Higuchi, and Korsmeyer-Peppas) were checked to determine drug release behavior. The Korsmeyer-Peppas mathematical model showed the best fit and rate-constant ***k***_KP_ of acidic pH 5.5 was found to be higher than that of physiological pH 7.4. The secondary parameters, Akaiki Information Constant (AIC) and Model Selection Criteria (MSC) were also checked to confirm the Korsmeyer-Peppas release model.

**Supplementary Figure 3.** Drug release from H40-NAG at physiological pH 7.4 and acidic pH 5.5 over 120 hours (5 days).

### Cellular uptake


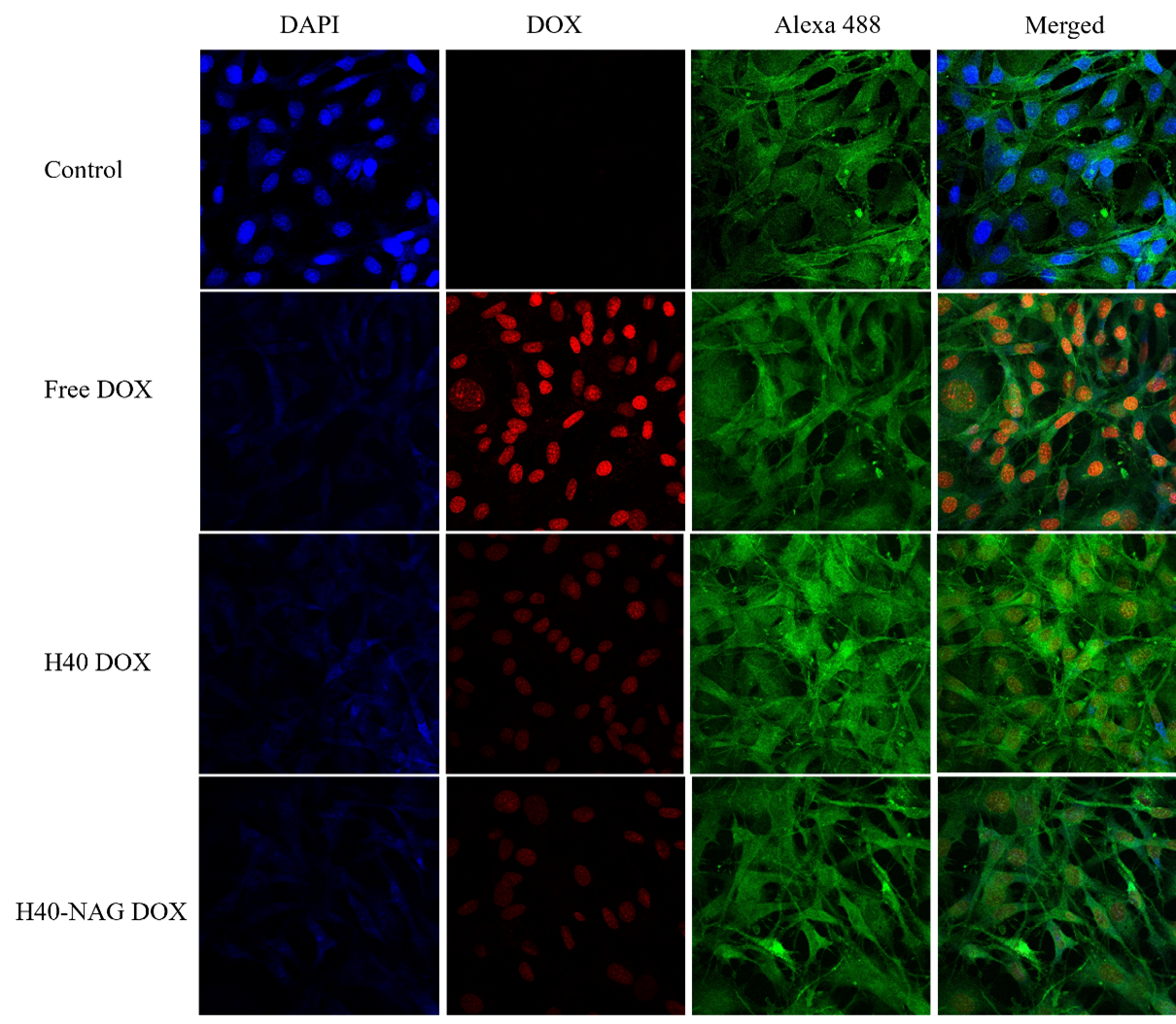


**Supplementary Figure 4.** Confocal images for NIH-3T3 cells treated with free DOX, H40-OH+DOX, and H40-NAG+DOX.


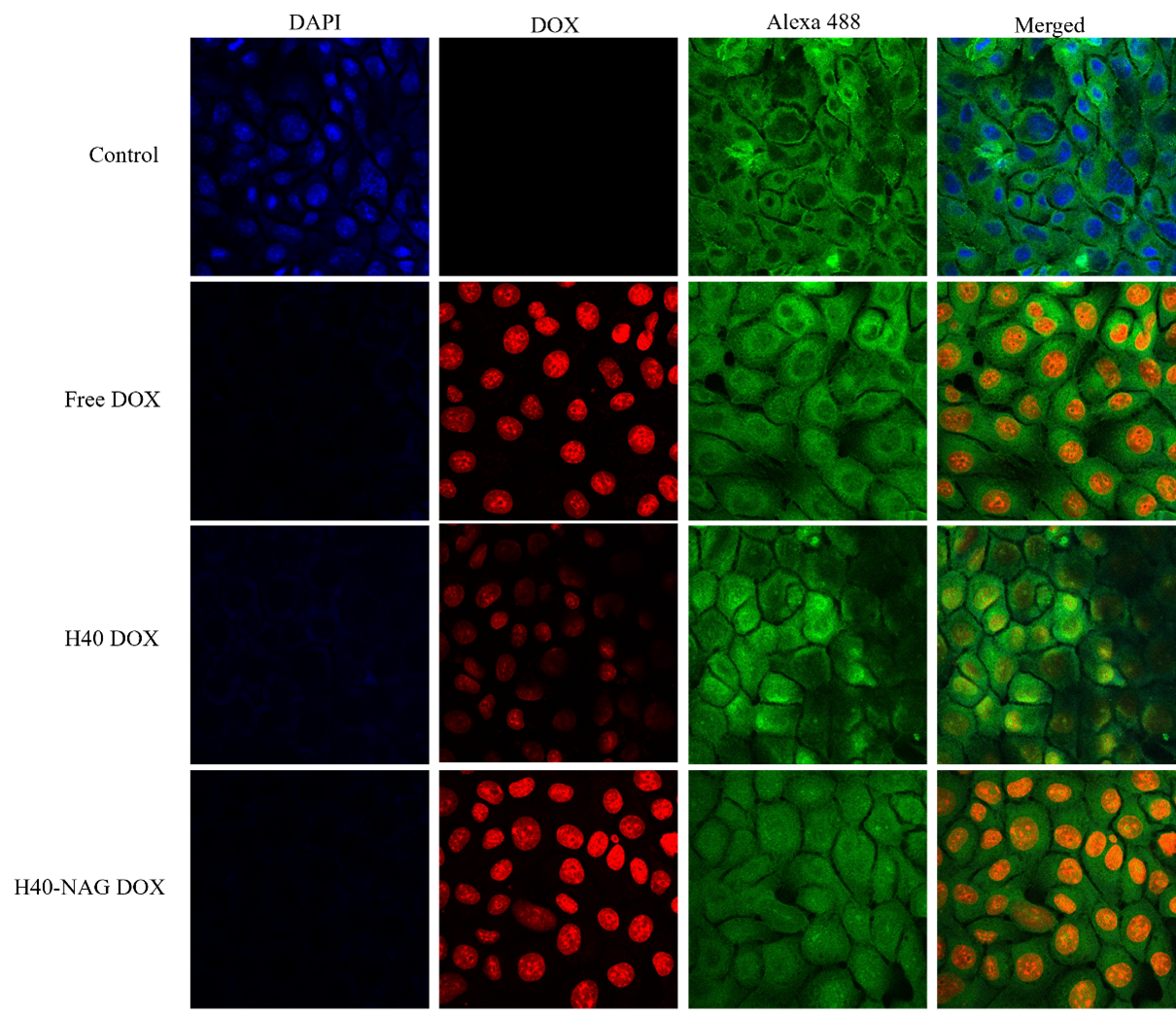


**Supplementary Figure 5.** Confocal images for 4T1 cells treated with free DOX, H40-OH+DOX, and H40-NAG+DOX.

### Cell cytotoxicity assessment


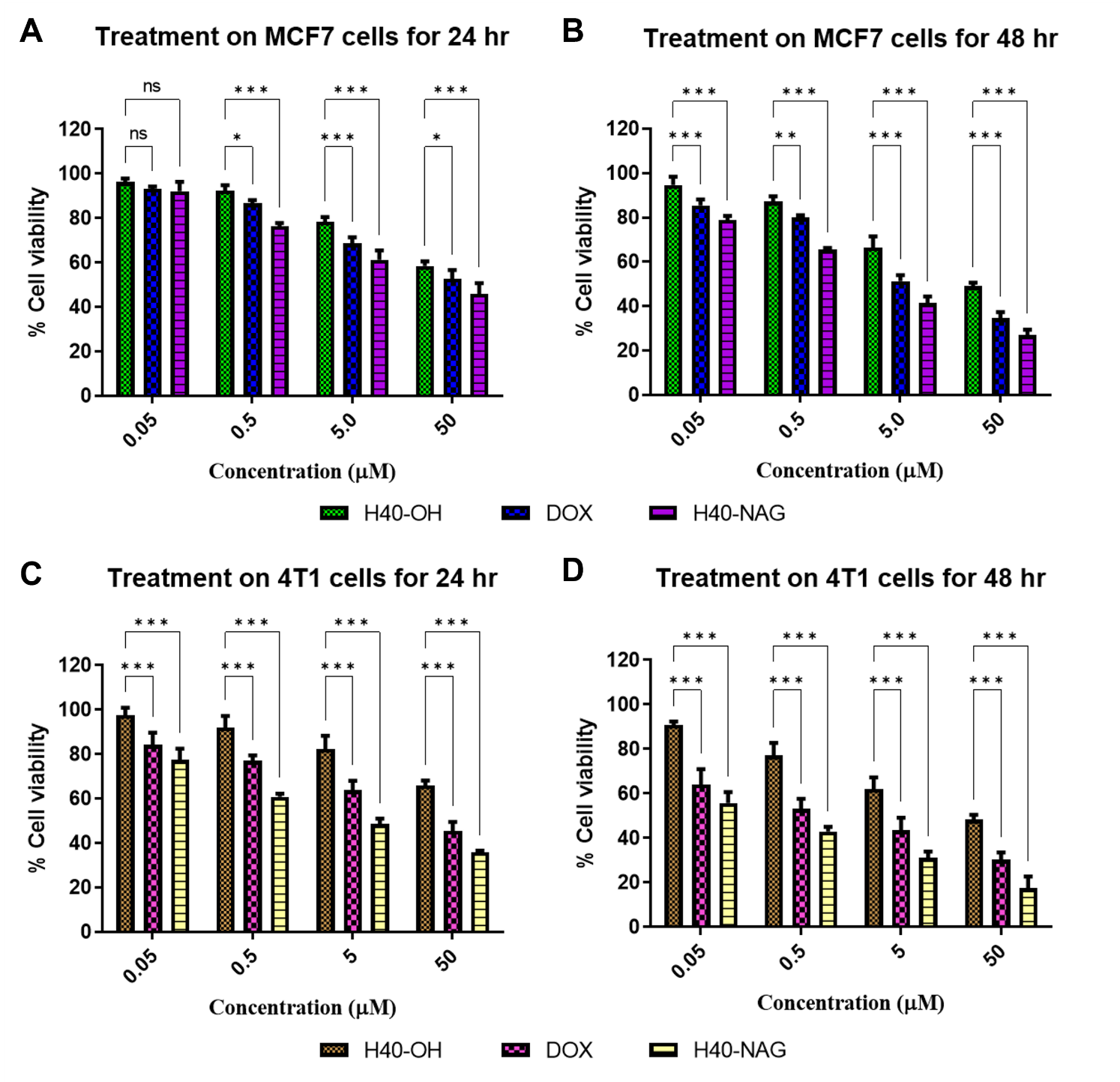


Supplementary Figure 6. Cytotoxicity of free DOX, DOX-loaded H40-OH and DOX-loaded H40-NAG in MCF7 (A and B) and 4T1 (C and D) cells at 24 and 48 hr. (*P<0.05, **P<0.002, **P<0.001 determined by Two-way ANOVA and Dunnett’s multiple comparison-test)

# References

Crowley, L. C., Marfell, B. J., Scott, A. P., & Waterhouse, N. J. (2016). Quantitation of apoptosis and necrosis by annexin V binding, propidium iodide uptake, and flow cytometry. *Cold Spring Harbor Protocols*, *2016*(11), 953–957. https://doi.org/10.1101/pdb.prot087288

Gajbhiye, V., Escalante, L., Chen, G., Laperle, A., Zheng, Q., Steyer, B., Gong, S., & Saha, K. (2014). Drug-loaded nanoparticles induce gene expression in human pluripotent stem cell derivatives. *Nanoscale*, *6*(1), 521–531. https://doi.org/10.1039/c3nr04794f

Kumar, P., Paknikar, K. M., & Gajbhiye, V. (2018). A robust pH-sensitive unimolecular dendritic nanocarrier that enables targeted anti-cancer drug delivery via GLUT transporters. *Colloids and Surfaces B: Biointerfaces*, *171*(July), 437–444. https://doi.org/10.1016/j.colsurfb.2018.07.053

Kumar, P., Tambe, P., Paknikar, K. M., & Gajbhiye, V. (2017). Folate/N-acetyl glucosamine conjugated mesoporous silica nanoparticles for targeting breast cancer cells: A comparative study. *Colloids and Surfaces B: Biointerfaces*, *156*, 203–212. https://doi.org/10.1016/j.colsurfb.2017.05.032

Salve, R., Haldar, N., & Shaikh, A. (2024). *H40-TEPA-PEG nanoconjugates for targeted siRNA-delivery and gene silencing in breast cancer cells*. *April*, 1–8. https://doi.org/10.3389/fbioe.2024.1383495

Yu, S., Dong, R., Chen, J., Chen, F., Jiang, W., Zhou, Y., Zhu, X., & Yan, D. (2014). Synthesis and self-assembly of amphiphilic aptamer-functionalized hyperbranched multiarm copolymers for targeted cancer imaging. *Biomacromolecules*, *15*(5), 1828–1836. https://doi.org/10.1021/bm5002203
